# External codes for multiple unicast networks via interference alignment

**DOI:** 10.1007/s10623-024-01439-1

**Published:** 2024-06-10

**Authors:** F. R. Kschischang, F. Manganiello, A. Ravagnani, K. Savary

**Affiliations:** 1 University of Toronto, Toronto, Canada; 2 Clemson University, Clemson, USA; 3 Eindhoven University of Technology, Eindhoven, The Netherlands

**Keywords:** Multiple unicast networks, Finite fields, External codes, Achievable rate region, Bound, 94A05

## Abstract

We introduce a formal framework to study the multiple unicast problem for a coded network in which the network code is linear over a finite field and fixed. We show that the problem corresponds to an interference alignment problem over a finite field. In this context, we establish an outer bound for the achievable rate region and provide examples of networks where the bound is sharp. We finally give evidence of the crucial role played by the field characteristic in the problem.

## Introduction

The typical scenario considered in the context of *network coding* consists of one or multiple sources of information attempting to communicate to multiple terminals through a network of intermediate nodes. Various communication paradigms have been studied in this setting, including noisy, adversarial, error-free multicast, and multiple-unicast networks; see [1–8] among many others.

To our best knowledge, in most network coding references users are allowed to freely design both the *network code* (i.e., how the intermediate vertices process information packets) and the *external codes* of the sources. In particular, designing the network code is part of the communication problem.

This paper considers the multiple unicast problem when the network code is linear and *fixed*, and only the external network codes can be freely designed by the source-receiver pairs. In this context, source-receiver pairs compete for network resources and interfere with each other. We argue that, in this regime, the multiple unicast problem corresponds to an *interference alignment problem* over finite fields. While interference alignment over the complex field has been extensively studied in more classical information theory settings, methods do not extend to finite fields in any obvious way. We refer to [9, 10] for the “classical” interference alignment problem.

The concept of interference alignment has also been considered over finite fields, in connection with network coding. For example, [6, 7] propose interference alignment solutions, in combination with network coding, to construct schemes for coded, multiple unicast networks. Related contributions are [9, 11–13], all of which study a problem that is different from the one we address in this paper.

This work makes three main contributions: (1) it introduces a framework for investigating multi-shot interference alignment problems over finite fields; (2) it establishes an outer bound for the achievable rate regions in the context outlined above and provides examples where the bound is sharp; (3) it shows how the field characteristic plays a crucial role in the solution to this problem. Note that (3) already played an important role in the networks introduced in [4], but is in sharp contrast with what is typically observed in network coding results for unicast networks, where the field *size*, rather than the characteristic, is the main player.

The rest of the paper is organized as follows. In Sect. 2 we formally introduce the communication model and formulate the problem on which we focus. In Sect. 3 we formalize the concepts of achievable rate regions, considering the number of channel uses. Section 4 establishes an outer bound for said achievable rate regions, and Sect. 5 describes the role played by the field characteristic. The paper contains several examples that illustrate concepts and results.

## Communication model and problem formulation

Throughout this paper, *q* is a prime power and $\mathbb {F}_q$ denotes the finite field with *q* elements. We denote the set of positive integers by $\mathbb {N}=\{1,2, \ldots \}$. All vectors in this paper are row vectors. For $\alpha _1,\dots ,\alpha _\ell \in \mathbb {F}_q^n$, we denote by $\langle \alpha _1,\dots ,\alpha _\ell \rangle$ the $\mathbb {F}_q$-span of $\alpha _1,\dots ,\alpha _\ell$. When $\mathbb {F}_p\subseteq \mathbb {F}_q$, we denote by $\langle \alpha _1,\dots ,\alpha _\ell \rangle _p$ the $\mathbb {F}_p$-span of $\alpha _1,\dots ,\alpha _\ell$.

We start by informally describing the problem studied in this paper, deferring rigorous definitions for later.

**Problem Formulation** We consider *n* uncoordinated sources and terminals, denoted by $S_1,...,S_n$ and $T_1,...,T_n$, respectively. Terminal $T_i$ is interested in decoding only the symbols emitted by source $S_i$ (*multiple unicast* problem). The sources are connected to the terminals via a network of intermediate nodes, $\mathcal {N}$ (a directed, acyclic multigraph). The alphabet of the network is $\mathbb {F}_q$ and each edge has a capacity of one symbol. Alphabet symbols combine linearly at the intermediate nodes of the network, i.e., *linear network coding* is used; see [2, 3].

We are interested in describing the region of achievable rates for a network of the type we just described, assuming that the operations performed by the intermediate nodes are linear and *fixed*. In other words, sources and terminals cannot change how the network’s nodes linearly combine symbols, but are free to agree on a codebook. Under these assumptions, source-receiver pairs compete for the network’s resources.

The following example illustrates the problem at hand.

### Example 1

**Fig. 1:**
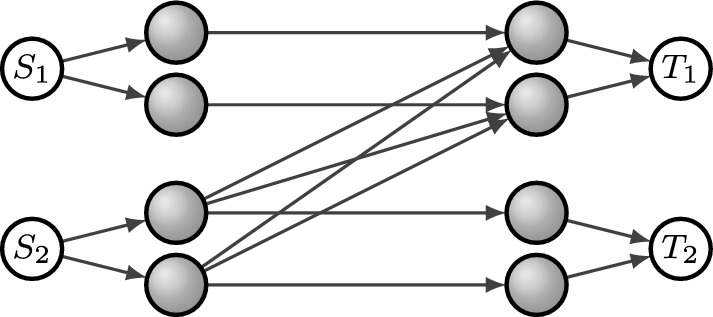
Network for Example 1

Fig. 1 depicts a network with two sources and terminals. Terminal $T_i$ is interested only in decoding messages from source $S_i$. The operations performed by the gray vertices are fixed. We will return to this network in Example 4.

In this paper, we propose the following formal definition of a communication network, which will facilitate the analysis of the problem we have described above.

### Definition 2

A **multiple unicast network** is a 4-tuple

$$\begin{aligned} \mathcal {N}=(\mathcal {V},\mathcal {E},(S_1, \ldots ,S_n), (T_1, \ldots ,T_n)), \end{aligned}$$

where:

(A) $(\mathcal {V},\mathcal {E})$ is a finite, directed, acyclic multigraph, which may include parallel edges;
(B) $n \ge 1$ is an integer;
(C) $S_1, \ldots ,S_n \in \mathcal {V}$ are distinct vertices called **sources**;
(D) $T_1, \ldots ,T_n \in \mathcal {V}$ are distinct vertices called **terminals** or **sinks**.

We also assume that the following hold:

(E) $$\{S_1, \ldots , S_n\} \cap \{T_1, \ldots , T_n\}=\emptyset $$;
(F) for any $i \in \{1,\ldots ,n\}$, there exists a directed path in $(\mathcal {V},\mathcal {E})$ connecting $S_i$ to $T_i$;
(G) sources do not have incoming edges and terminals do not have outgoing edges;
(H) for every vertex $V \in \mathcal {V}$, there exists a directed path from $S_i$ to *V* and from *V* to $T_j$, for some $i,j \in \{1, \ldots ,n\}$.

For $V\in \mathcal {V}$ we denote by $\partial ^-(V)$, respectively $\partial ^+(V)$, the indegree, respectively outdegree, of *V*, meaning the number of edges incident to, respectively from, *V*. Moreover, let $\mathcal {V}^*=\mathcal {V}\setminus (\{S_1, \ldots ,S_n\} \cup \{T_1, \ldots , T_n\})$ denote the set of nonsource and nonterminal vertices, meaning the set of intermediate network nodes.

We assume that the intermediate nodes of a network process the alphabet symbols linearly. This is made rigorous by the following concept.

### Definition 3

Let $\mathcal {N}$ be as in Definition 2. A **linear network code**, or simply **network code**, for $\mathcal {N}$ is a tuple of matrices

$$\begin{aligned} \mathcal {F}=\left( \mathcal {F}_V \in \mathbb {F}_q^{\partial ^-(V) \times \partial ^+(V)}\mid V \in \mathcal {V}^*\right) . \end{aligned}$$

Given a network code, the network operates as follows. Fix an intermediate vertex $V\in \mathcal {V}^*$ and let $a=\partial ^-(V)$, $b=\partial ^+(V)$ be the indegree and outdegree of *V*, respectively. Then *V* collects a vector of alphabet symbols $(x_1, \ldots , x_a)$ over the incoming edges, and emits the entries of

$$\begin{aligned} \begin{pmatrix} y_1&\ldots&y_b \end{pmatrix}= \begin{pmatrix} x_1&\ldots&x_a \end{pmatrix} \cdot \mathcal {F}_V \in \mathbb {F}_q^b \end{aligned}$$

over the outgoing edges. This fully specifies how *V* processes information, provided a linear extension of the partial order of the network edges is fixed. Thus, $x_1$ is the symbol that arrives on the minimum edge (with respect to this linear order) at *V*, $x_2$ is the symbol that arrives on the minimum of the remaining edges, etc., and similarly for $y_1, \dots , y_b$. In this paper, networks are delay-free, and communication is instantaneous.

### Example 4

**Fig. 2:**
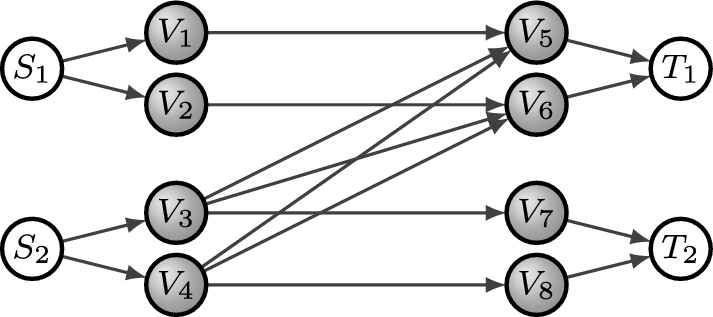
Network from Example 1 with labeled vertices

The same network admits multiple network codes. For this example, consider the network $\mathcal {N}$ from Example 1 with the intermediate nodes labeled as in Fig. 2 and let us assume that the alphabet is $\mathbb {F}_3$.

The following are two network codes defined on the network of Fig. 2 where the order of the entries in the tuple follows the order of the intermediate nodes, and the order of the entries in the matrices agrees with the order of the outgoing and incoming channels in the nodes respectively.

$$\begin{aligned} \mathcal {F}_1=\left( \begin{pmatrix} 1 \end{pmatrix}, \begin{pmatrix} 1 \end{pmatrix}, \begin{pmatrix} 1&1&1 \end{pmatrix}, \begin{pmatrix} 1&1&1 \end{pmatrix}, \begin{pmatrix} 1\\ 1\\ 1 \end{pmatrix}, \begin{pmatrix} 1\\ 1\\ 1 \end{pmatrix}, \begin{pmatrix} 1 \end{pmatrix}, \begin{pmatrix} 1 \end{pmatrix}\right) \end{aligned}$$

$$\begin{aligned} \mathcal {F}_2=\left( \begin{pmatrix} 1 \end{pmatrix}, \begin{pmatrix} 1 \end{pmatrix}, \begin{pmatrix} 1&1&1 \end{pmatrix}, \begin{pmatrix} 1&1&1 \end{pmatrix}, \begin{pmatrix} 1\\ 1\\ 1 \end{pmatrix}, \begin{pmatrix} 1\\ 1\\ 2 \end{pmatrix}, \begin{pmatrix} 1 \end{pmatrix}, \begin{pmatrix} 1 \end{pmatrix}\right) \end{aligned}$$

The choice of a linear network code $\mathcal {F}$ for a communication network $\mathcal {N}$ induces end-to-end transfer matrices, one for each source-terminal pair. We denote by $F_{i,j}$ the transfer matrix from $S_i$ to $T_j$.

### Example 5

Using the network $\mathcal {N}$ from Example 1, the network code $\mathcal {F}_1$ induces the transfer matrices

$$\begin{aligned} F_{1,1}=F_{2,2}= \begin{pmatrix} 1&{}0\\ 0&{}1 \end{pmatrix},\ F_{1,2} = \begin{pmatrix} 0&{}0\\ 0&{}0 \end{pmatrix},\ \text {and} \ F_{2,1}= \begin{pmatrix} 1&{}1\\ 1&{}1 \end{pmatrix}, \end{aligned}$$

whereas the network code $\mathcal {F}_2$ induces transfer matrices

$$\begin{aligned} F_{1,1}=F_{2,2}= \begin{pmatrix} 1&{}0\\ 0&{}1 \end{pmatrix},\ F_{1,2} = \begin{pmatrix} 0&{}0\\ 0&{}0 \end{pmatrix},\ \text {and}\ F_{2,1}= \begin{pmatrix} 1&{}1\\ 1&{}2 \end{pmatrix}. \end{aligned}$$

In one channel use, assuming that $S_i$ transmits $x_i\in \mathbb {F}_q^{\partial ^+(S_i)}$, terminal $T_j$ observes the vector

$$\begin{aligned} y_j=\sum _{i=1}^nx_iF_{i,j}\in \mathbb {F}_q^{\partial ^-(T_j)}. \end{aligned}$$

Since in this paper both the communication network $\mathcal {N}$ and the linear network code $\mathcal {F}$ are supposed to be *fixed*, the end-to-end transfer matrices induced by them fully specify the communication channel. We, therefore, propose the following definition.

### Definition 6

A *q* **-ary linear multiple unicast channel** (in short, *q* **-LMUC**) is a 4-tuple $\mathcal {L}=(n,\pmb {s},\pmb {t},F)$, where $n\in \mathbb {N}$ is a positive integer, $\pmb {s}=(s_1,\dots ,s_n),\pmb {t}=(t_1,\dots ,t_n)\in \mathbb {N}^n$, and $F\in \mathbb {F}_q^{s\times t}$, where $s=\sum _{i=1}^n s_i$ and $t=\sum _{i=1}^n t_i$. We call *F* the **transfer matrix** and regard it as a block matrix

$$\begin{aligned} F=\begin{pmatrix}F_{1,1}&{} \cdots &{} F_{1,n} \vdots &{}\ddots &{}\vdots \\ F_{n,1}&{}\cdots &{}F_{n,n} \end{pmatrix}, \end{aligned}$$

where block $F_{i,j}$ has size $s_i \times t_j$.

Here *n* represents the number of source-terminal pairs, $s_i=\partial ^+(S_i)$, and $t_i=\partial ^-(T_i)$ for $i=1,\dots ,n$. Moreover, *F* collects the transfer matrices for each source-terminal pair. Note that we do not need to remember the communication network or the entire network code. The matrix *F* fully describes the end-to-end channel laws for a single channel use.

We can extend this definition for multiple uses of the channel as follows. Suppose that the network is used $m \ge 1$ times. The channel input is an element $x=(x_1,\dots ,x_n)\in \mathbb {F}_{q^m}^{s_1}\times \cdots \times \mathbb {F}_{q^m}^{s_n}=\mathbb {F}_{q^m}^s$. More precisely, for all $i \in \{1,\dots ,n\}$, $x_i\in \mathbb {F}_{q^m}^{s_i}$ is the input that source $S_i$ emits. Then the channel output is $\smash {y=(y_1,\dots ,y_n)\in \mathbb {F}_{q^m}^{t_1}\times \cdots \times \mathbb {F}_{q^m}^{t_n}=\mathbb {F}_{q^m}^t}$, where

1 $$\begin{aligned} y_i=x_iF_{i,i}+\sum _{j\ne i}x_jF_{j,i} \end{aligned}$$

is the vector that terminal $T_i$ receives on its incoming edges. Note that the field extension $\mathbb {F}_{q^m}$ models *m* uses of the channel because the network code is assumed to be $\mathbb {F}_q$-linear.

### Remark 7

Given any *q*-LMUC as in Definition 6, it is always possible to construct a multiple unicast network $\mathcal {N}$ and a network code $\mathcal {F}$ for $\mathcal {N}$ that induces the given transfer matrix. We illustrate the procedure with an example.

### Example 8

The 11-LMUC

$$\begin{aligned} \mathcal {L}= \left( 2,(1,2),(2,2), \begin{pmatrix} 1 &{} 0 &{} 2 &{} 3\\ 0 &{} 4 &{} 5 &{} 0\\ 6 &{} 7 &{} 0 &{} 0 \end{pmatrix}\right) \end{aligned}$$

induces the matrices

$$\begin{aligned} F_{1,1}= \begin{pmatrix} 1&0 \end{pmatrix},\quad F_{1,2}= \begin{pmatrix} 2&3 \end{pmatrix},\quad F_{2,1}= \begin{pmatrix} 0 &{} 4\\ 6 &{} 7 \end{pmatrix},\quad F_{2,2}= \begin{pmatrix} 5&{}0\\ 0&{}0 \end{pmatrix}. \end{aligned}$$

**Fig. 3:**
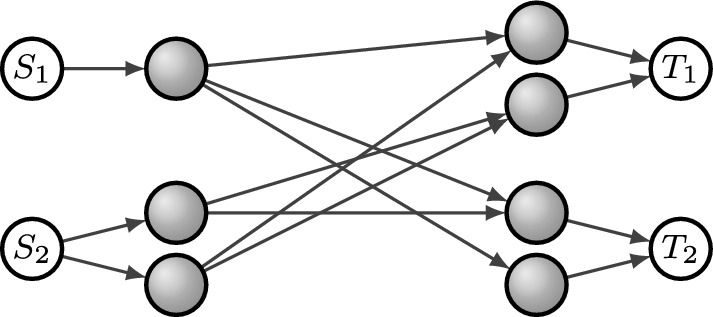
Network for Example 8

A network code that induces these matrices is

$$\begin{aligned} \mathcal {F}=\left( \begin{pmatrix} 1&1&1 \end{pmatrix}, \begin{pmatrix} 1&1 \end{pmatrix}, \begin{pmatrix} 1&1 \end{pmatrix}, \begin{pmatrix} 1 \\ 6 \end{pmatrix}, \begin{pmatrix} 4 \\ 7 \end{pmatrix}, \begin{pmatrix} 2 \\ 5 \end{pmatrix}, \begin{pmatrix} 3 \end{pmatrix}\right) , \end{aligned}$$

realized by the network depicted in Fig. 3. Note that

$$\begin{aligned} \mathcal {F}=\left( \begin{pmatrix} 1&2&3 \end{pmatrix}, \begin{pmatrix} 4&5 \end{pmatrix}, \begin{pmatrix} 6&7 \end{pmatrix}, \begin{pmatrix} 1 \\ 1 \end{pmatrix}, \begin{pmatrix} 1 \\ 1 \end{pmatrix}, \begin{pmatrix} 1 \\ 1 \end{pmatrix}, \begin{pmatrix} 1 \end{pmatrix}\right) , \end{aligned}$$

also induces the given transfer matrix.

Given a *q*-LMUC $\mathcal {L}=(n,\pmb {s},\pmb {t},F)$ representing a *q*-ary linear multiple unicast channel, we are interested in determining which rates can be achieved by carefully selecting the codebooks of the sources. Recall that each terminal $T_i$ is interested in decoding *only* its corresponding source $S_i$. Therefore the information emitted by the sources $S_j$ with $j \ne i$ acts as interference for terminal $T_i$.

## Achievable rate regions and their properties

In this section, we propose a formal framework to describe the capacity of a *q*-LMUC, based on the concept of *fan-out set* and *unambiguous code*. We start with the concept of code(book).

### Definition 9

Let $\mathcal {L}=(n,\pmb {s},\pmb {t},F)$ be a *q*-LMUC and let $m \ge 1$ be an integer. An *m* **-external code**, or simply an *m* **-code**, for $\mathcal {L}$ is a Cartesian product $C=C_1\times \cdots \times C_n$, where $C_i \subseteq \mathbb {F}_{q^m}^{s_i}$ for all $i \in \{1,\ldots ,n\}$. The elements of each $C_i$ are called **codewords**.

Note that we do *not* require that *C* is a linear space. Recall that the parameter *m* in Definition 9 represents the number of channel uses, as already mentioned when introducing Eq. (1). In the sequel, for a *q*-LMUC $\mathcal {L}=(n,\pmb {s},\pmb {t},F)$ and for $i\in \{1,\dots ,n\}$, we denote by $\pi _i:\mathbb {F}_{q^m}^t\rightarrow \mathbb {F}_{q^m}^{t_i}$ the projection on the *i*th block of coordinates (recall that $\pmb {t}=(t_1, \ldots ,t_n)$ and $t=t_1 + \cdots + t_n$).

### Definition 10

Let $\mathcal {L}=(n,\pmb {s},\pmb {t},F)$ be a *q*-LMUC, $i \in \{1, \ldots , n\}$, *C* an *m*-code for $\mathcal {L}$, and $x \in C_i$. We denote by

$$\begin{aligned} \text {Fan}_i(x,C):=\{\pi _i((x_1,\dots ,x_{i-1},x,x_{i+1},\dots , x_n)F) \mid x_j\in C_j \text{ for } \text{ all } j \ne i\} \end{aligned}$$

the *i* **-th fan-out set** of *x* with respect to terminal *i* and the code *C*. The *i* **-th fan-out set** of *C* is $\text {Fan}_i(C)=\cup _{x\in C_i}{\text {Fan}_i(x,C)}\subseteq \mathbb {F}_{q^m}^{t_i}$, for all $i \in \{1,\ldots ,n\}$.

Following the notation of Definition 10, $\text {Fan}_i(x,C)$ is the set of possible words that the *i*th terminal can possibly receive when the *i*th source emits *x* and the other sources emit their own codewords. Fan-out sets relate to the concept of interference as follows.

### Definition 11

Let $\mathcal {L}$, *m*, and *C* be as in Definition 9. We define the **interference set** of *C* at terminal $T_i$ as

$$\begin{aligned} \text {IS}_i(C)=\text {Fan}_i(0,C)=\left\{ \sum _{j\ne i}c_jF_{j,i}\mid c_j\in C_j\right\} . \end{aligned}$$

### Remark 12

Note that using Definition 11 we can rewrite Eq. (1) as

2 $$\begin{aligned} \text {Fan}_i(x,C)=xF_{i,i} +\text {IS}_i(C) = \{xF_{i,i} + y \mid y \in \text {IS}_i(C)\}. \end{aligned}$$

for any *m*-code *C*, any $i \in \{1, \ldots , n\}$, and any $x \in C_i$. Therefore the *i*-th fan-out set of $x \in C_i$ is a **translate** (or a **coset**, if each $C_i$ is linear) of the interference set $\text {IS}_i(C)$.

Communication is considered to be successful when each codeword can be uniquely decoded. The following definition models this concept.

### Definition 13

Let $\mathcal {L}$, *m*, and *C* be as in Definition 9. We say that *C* is **unambiguous** for $\mathcal {L}$ if for all $i \in \{1,...,n\}$ and for all codewords $x_1,x_2 \in C_i$ with $x_1 \ne x_2$, we have $\text {Fan}_i(x_1,C) \cap \text {Fan}_i(x_2,C) = \emptyset$.

An unambiguous code, as in the previous definition, uniquely defines decoder maps. More precisely, the *i* **-th decoder** is the map $D_i:\text {Fan}_i(C)\rightarrow \mathbb {F}_{q^m}^{s_i}$ defined by $D_i(v_i)=x_i$ for all $v_i \in \text {Fan}_i(C)$, where $x_i \in C_i$ is the only element with $v_i \in \text {Fan}_i(x_i,C)$.

### Remark 14

Following the notation of Definition 13, if *C* is an unambiguous *m*-code for $\mathcal {L}$ and $C'$ is an *m*-code for $\mathcal {L}$ with $C' \subseteq C$, then $C'$ is unambiguous as well.

We are now ready to give a rigorous definition of *the achievable rate region* of a *q*-LMUC $\mathcal {L}$. For convenience, for $m \ge 1$ we define the set

$$\begin{aligned} \log _{q^m}(\mathbb {N}^n)=\{(\log _{q^m}(u_1),...,\log _{q^m}(u_n)) \mid (u_1,...,u_n) \in \mathbb {N}^n\}. \end{aligned}$$

### Definition 15

The *m* **-shot achievable rate region** of a *q*-LMUC $\mathcal {L}$ as in Definition 6 is the set

$$\begin{aligned} \mathcal {R}_m(\mathcal {L})=\{\alpha \in \log _{q^m}(\mathbb {N}^n) \mid \exists \, C=C_1\times \cdots \times C_n \text{ unambiguous } \text{ m-code } \text{ for } \mathcal {L} \\ \text{ with } \log _{q^m}(|C_i|) = \alpha _i \ \forall \ 1 \le i \le n \} \subseteq \mathbb {R}_{\ge 0}^n. \end{aligned}$$

The **achievable rate region** of $\mathcal {L}$ is the set

$$\begin{aligned} \mathcal {R}(\mathcal {L}) = \overline{\bigcup _{m \ge 1} \mathcal {R}_m(\mathcal {L})}, \end{aligned}$$

where the overline indicates the closure operator with respect to the Euclidean topology on $\mathbb {R}^n$. The elements of $\mathcal {R}(\mathcal {L})$ elements are called **achievable rates**.

The following example illustrates that different LMUCs supported on the same network might have very different achievable rate regions.

### Example 16

The two 3-LMUCs induced by Example 4 are

$$\begin{aligned} \mathcal {L}_1=\left( 2,(2,2),(2,2), \begin{pmatrix} 1 &{} 0 &{} 0 &{} 0\\ 0 &{} 1 &{} 0 &{} 0\\ 1 &{} 1 &{} 1 &{} 0\\ 1 &{} 1 &{} 0 &{}1 \end{pmatrix}\right) , \ \ \mathcal {L}_2=\left( 2,(2,2),(2,2), \begin{pmatrix} 1 &{} 0 &{} 0 &{} 0\\ 0 &{} 1 &{} 0 &{} 0\\ 1 &{} 1 &{} 1 &{} 0\\ 1 &{} 2 &{} 0 &{}1 \end{pmatrix}\right) . \end{aligned}$$

Although the two 3-LMUC have the same underlying network, they have different 1-shot achievable rate regions. Indeed, for $\mathcal {L}_1$, the code $\langle (1,2)\rangle _{\mathbb {F}_3}\times \mathbb {F}_3^2$ is unambiguous, meaning that $(1,2)\in \mathcal {R}_1(\mathcal {L})$. On the other hand, $(1,2)\notin \mathcal {R}_1(\mathcal {L}_2)$, as we now briefly explain. In $\mathcal {L}_2$, the matrix $F_{2,1}$ is invertible. Moreover, a rate of the form $(\alpha _1,2)$ can be achieved only by a code of the form $C=C_1 \times \mathbb {F}_3^2$. Together with the invertibility of $F_{2,1}$, this implies that $\text {IS}_1(C)=\mathbb {F}_3^2$, making $C=\{0\}\times \mathbb {F}_3^2$ the only unambiguous code with $C_2=\mathbb {F}_3^2$.

The following result states a quite intuitive property of achievable rate regions.

### Proposition 17

Let $\mathcal {L}$ be a *q*-LMUC as in Definition 6 and let $m \ge 1$. If $(\alpha _1, \ldots , \alpha _n) \in \mathcal {R}_m(\mathcal {L})$ and $\smash {(\beta _1, \ldots ,\beta _n) \in \log _{q^m}(\mathbb {N}^n)}$ satisfies $\beta _i \le \alpha _i$ for all $i \in \{1, \ldots , n\}$, then we have $(\beta _1, \ldots , \beta _n) \in \mathcal {R}_m(\mathcal {L})$.

### Proof

Since $(\alpha _1, \ldots , \alpha _n) \in \mathcal {R}_m(\mathcal {L})$, there exists an *m*-code $C=C_1 \times \cdots \times C_n$ unambiguous for $\mathcal {L}$ with $|C_i|=q^{m \alpha _i}$ for all *i*. Since $(\beta _1, \ldots , \beta _n) \in \log _{q^m}(\mathbb {N}^n)$, for all *i* there exists $D_i \subseteq C_i$ with $|D_i|=q^{m \beta _i}$. By Remark 14, the *m*-code $D=D_1 \times \cdots \times D_n$ is unambiguous for $\mathcal {L}$. This establishes the desired result. $\square$

We now investigate how the various achievable rate regions relate to each other. The following two results are inspired by (but do not immediately follow from) the concept of time sharing.

### Proposition 18

Let $\mathcal {L}$ be a *q*-LMUC as in Definition 6 and let $m,m' \ge 1$. We have

$$\begin{aligned} \mathcal {R}_{m+m'}(\mathcal {L}) \supseteq \frac{m\, \mathcal {R}_m(\mathcal {L}) + m' \, \mathcal {R}_{m'}(\mathcal {L})}{m+m'}. \end{aligned}$$

### Proof

Let *C* and $C'$ be an *m*-code and an $m'$-code, respectively, for $\mathcal {L}$. Let $\{\gamma _1,...,\gamma _m\}$ and $\smash {\{\gamma '_1,...,\gamma '_{m'}\}}$ be ordered bases of $\smash {\mathbb {F}_{q^m}}$ and $\smash {\mathbb {F}_{q^{m'}}}$ over $\mathbb {F}_q$, respectively. We denote by $\smash {\varphi _m:\mathbb {F}_q^m \rightarrow \mathbb {F}_{q^m}}$ the $\mathbb {F}_q$-isomorphism $(a_1,\ldots ,a_m) \mapsto \sum _{i=1}^m a_i \gamma _i$. Define $\smash {\varphi _{m'}:\mathbb {F}_q^{m'} \rightarrow \mathbb {F}_{q^{m'}}}$ analogously. Fix an ordered basis $\smash {\{\beta _1, \ldots ,\beta _{m+m'}\}}$ of $\mathbb {F}_{q^{m+m'}}$ over $\mathbb {F}_q$. We extend these three $\mathbb {F}_q$-linear maps coordinate-wise. Take

$$\begin{aligned} D:= \varphi _m^{-1}(C) \times \varphi _{m'}^{-1}(C'). \end{aligned}$$

Then the $(m+m')$-code $\varphi _{m+m'}(D)$ is unambiguous and it satisfies

$$\begin{aligned} \log _{q^{m+m'}} \left( \varphi _{m+m'}(D) \right) = \frac{m}{m+m'} \log _{q^m}(|C|) + \frac{m'}{m+m'} \log _{q^{m'}}(|C'|). \end{aligned}$$

This establishes the desired result. $\square$

The following result shows that $\mathcal {R}(\mathcal {L})$ contains the convex hull of $\mathcal {R}_1(\mathcal {L})$.

### Theorem 19

Let $\mathcal {L}$ be a *q*-LMUC as in Definition 6. Denote by $\text{ conv }(\mathcal {R}_1(\mathcal {L}))$ the convex hull of $\mathcal {R}_1(\mathcal {L})$, i.e., the set of convex combinations of the points of $\mathcal {R}_1(\mathcal {L})$. Then

$$\begin{aligned} \mathcal {R}(\mathcal {L}) \supseteq \text{ conv }(\mathcal {R}_1(\mathcal {L})). \end{aligned}$$

### Proof

Define $Z=|\mathcal {R}_1(\mathcal {L})|$ and let $\mathcal {R}_1(\mathcal {L})=\{\alpha ^{(\ell )} \mid 1 \le \ell \le Z\}$. Let $A:=\{(a_1,...,a_Z) \in [0,1]^Z \mid a_1+ \cdots +a_Z=1\}$, and let $a \in A$. We will show that for any real number $\varepsilon >0$, there exists $m \ge 1$ and $\beta \in \mathcal {R}_m(\mathcal {L})$ with

$$\begin{aligned} \left\| \sum _{\ell =1}^N a_\ell \alpha ^{(\ell )} -\beta \right\| \le \varepsilon . \end{aligned}$$

This will imply the desired theorem by the definition of closure in the Euclidean topology.

Since the result is clear if $\mathcal {R}_1(\mathcal {L})=\{(0,\dots ,0)\}$, we shall assume $\nu := \max _\ell \left\| \alpha ^{(\ell )} \right\| >0$. For all $\ell \in \{1, \dots , Z\}$, fix an unambiguous 1-code $\smash {C^{(\ell )} = C^{(\ell )}_1 \times \cdots \times C^{(\ell )}_n}$ that achieves rate $\alpha _\ell$, i.e., such that

$$\begin{aligned} \log _q \left( C^{(\ell )}_i \right) =\alpha _i \text{ for } \text{ all } \text{ i. } \end{aligned}$$

Since $A \cap \mathbb {Q}^n$ is dense in *A*, there exists a sequence $(s^k)_{k\in \mathbb {N}}\subseteq A \cap \mathbb {Q}^Z$ with the property that $\lim _{k\rightarrow \infty } s^k=a$. There exists $k_\varepsilon \in \mathbb {N}$ such that $\left\| s^k-a \right\| \le \varepsilon /(\nu \sqrt{Z})$ for all $k \ge k_\varepsilon$. Let $s=s^{k_\varepsilon }$ and $\smash {\beta = \sum _{\ell =1}^Z s_\ell \alpha _\ell }$ and observe that

$$\begin{aligned} \left\| \sum _{\ell =1}^Z a_\ell \alpha _\ell -\beta \right\| \le \nu \sum _{\ell =1}^Z (s_\ell -a_\ell ) \le \nu \sum _{\ell =1}^Z |s_\ell -a_\ell | \le \nu \sqrt{Z} \left\| s-a \right\| \le \varepsilon . \end{aligned}$$

Therefore, it suffices to show that $\beta \in \mathcal {R}_m(\mathcal {L})$ for some $m \ge 1$. Write $s=(s_1,...,s_Z)=(b_1/c_1,...,b_Z/c_Z)$, where the $b_\ell$’s and the $c_\ell$’s are integers, and let $m=c_1 \cdots c_Z$. Then $\beta$ can be achieved in *m* rounds by using code $C^{(\ell )}$ for $m b_\ell /c_\ell$ rounds, $\ell \in \{1,...,Z\}$, in any order. A more precise formulation can be obtained using field extension maps as in the proof of Proposition 18 (we do not go into the details). $\square$

## An outer bound for the achievable rate region

In this section, we establish an outer bound for the achievable rate region of a *q*-LMUC. Our proof technique derives a lower bound for the size of the fan-out sets introduced in Definition 10, which we obtain by estimating the “amount” of interference that the users cause to each other. The outer bound is stated in Theorem 24, which relies on two preliminary results.

### Remark 20

Let $M \in \mathbb {F}_q^{a \times b}$ be any matrix. Embed $\mathbb {F}_q$ into $\mathbb {F}_{q^m}$, where $m \ge 1$. It is well-known that the $\mathbb {F}_q$-rank of *M* is the same as its $\mathbb {F}_{q^m}$-rank.

We start with the following simple observation.

### Proposition 21

Let $\mathcal {L}=(n,\pmb {s},\pmb {t},F)$ be a *q*-LMUC and let $m \ge 1$ be an integer. If $n=1$, then $\mathcal {R}_m(\mathcal {L})=\{\alpha \in \log _q(\mathbb {N}) \mid 0 \le \alpha \le {\text {rank}}F\}$.

### Proof

Let $C\subseteq \mathbb {F}_{q^m}^{s_1}$ be an *m*-code. Then *C* is unambiguous if and only if $F(x)\ne F(y)$ for all $x,y\in C$ with $x\ne y$, i.e., if and only if the elements of *C* belong to distinct equivalent classes of $\mathbb {F}_{q^m}^{r_1}/\ker (F)$. This shows that if *C* is unambiguous, then $|C| \le q^{m \cdot {\text {rank}}(F)}$. Vice versa, taking one representative for each class of $\smash {\mathbb {F}_{q^m}^{r_1}/\ker (F)}$ produces an unambiguous *m*-code. $\square$

In the remainder of the section, we show how the argument in the previous proposition extends to an arbitrary number of users. We will need the following preliminary result.

### Lemma 22

Let *V*, *W* be linear spaces over $\mathbb {F}_{q^m}$, $m \ge 1$, and let $L:V \rightarrow W$ be an $\mathbb {F}_{q^m}$-linear map. For all non-empty sets $A \subseteq V$, we have $|L(A)| \cdot |\ker (L)| \ge |A|$.

### Proof

Define an equivalence relation on *A* by setting $a \sim b$ if and only if $L(a)=L(b)$, i.e., if and only if $a-b \in \ker (L)$. Then we know from elementary set theory that $|A/_\sim | = |f(A)|$. The equivalence class of $a \in A$ is $(a+\ker (L)) \cap B$ and, therefore, it has size at most $|\ker (L)|$. Since the equivalence classes partition *A*, we have $|A| \le |A/_\sim | \cdot |\ker (L)|$. This concludes the proof. $\square$

We are now ready to state the main result of this section, providing an outer bound for the achievable rate region of a *q*-LMUC.

### Notation 23

In the sequel, for a non-empty subset $I \subseteq \{1,...,n\}$ and $j \in I$, we denote by $F_{I,j}$ the submatrix of *F* formed by the blocks indexed by (*i*, *j*), as *i* ranges over *I*.

The main result of this section is the following outer bound.

### Theorem 24

Let $\mathcal {L}=(n,\pmb {s},\pmb {t},F)$ be a *q*-LMUC, and let $m \ge 1$ be an integer. Let $(\alpha _1,...,\alpha _n) \in \mathcal {R}_m(\mathcal {L})$. Then for all non-empty $I \subseteq \{1,\ldots ,n\}$ and for all $j \in I$, we have

$$\begin{aligned} \sum _{i \in I} \alpha _i \le {\text {rank}}(F_{I,j}) - {\text {rank}}(F_{I \setminus \{j\},j})+ \sum _{\begin{array}{c} k \in I \\ k \ne j \end{array}} s_k. \end{aligned}$$

Therefore, for all non-empty $I\subseteq \{1,\dots , n\}$, we have

$$\begin{aligned} \sum _{i \in I} \alpha _i \le \min _{j \in I} \left\{ {\text {rank}}(F_{I,j}) -{\text {rank}}(F_{I \setminus \{j\},j})+ \sum _{\begin{array}{c} k \in I \\ k \ne j \end{array}} s_k \right\} . \end{aligned}$$

### Proof

The result easily follows the definitions if $|I|=1$. Now fix an index set with $|I| \ge 2$, a tuple $(\alpha _1,...,\alpha _n) \in \mathcal {R}_m(\mathcal {L})$, and an unambiguous *m*-code $C=(C_1,\dots ,C_n)$ with $\alpha _i=\log _{q^m}(|C_i|)$ for all *i*. For $i \notin I$, we replace $C_i$ with an arbitrary subset of cardinality one. The resulting code is unambiguous by Remark 14, and it has

$$\begin{aligned} \log _{q^m}(|C_i|)= {\left\{ \begin{array}{ll} \alpha _i &{} \text{ if } i \in I\text{, } \\ 0 &{} \text{ if } i \notin I\text{. } \end{array}\right. } \end{aligned}$$

The remainder of the proof uses the following lower bound, which we will establish later.

### Claim A

For all $j \in I$ and $x \in C_j$, we have

3 $$\begin{aligned} |\text{ Fan}_j(x,C)| \ge \frac{\prod _{k \in I\setminus \{j\}}|C_k|}{|\ker F_{I \setminus \{j\},j}|}. \end{aligned}$$

Note that by fixing $j \in I$ and summing the inequality in Claim A over all $x \in C_j$ one obtains

4 $$\begin{aligned} \sum _{x \in C_j} |\text {Fan}_j(x,C)| \ge \frac{\prod _{k \in I}|C_k|}{|\ker F_{I \setminus \{j\},j}|} \quad \text{ for } \text{ all } j \in I\text{. } \end{aligned}$$

Since *C* is unambiguous, we have

$$\begin{aligned} \sum _{x \in C_j} |\text {Fan}_j(x,C)| = \left| \bigcup _{x \in C_j} \text {Fan}_j(x,C)\right| \le q^{m \cdot {\text {rank}}(F_{I,j})}, \end{aligned}$$

where the latter inequality follows from the fact that $\bigcup _{x \in C_j} \text {Fan}_j(x,C)$ is contained in the image of $F_{I,j}$. Therefore, the inequality in Eq. (4) implies

5 $$\begin{aligned} q^{m \cdot {\text {rank}}(F_{I,j})} \ge \frac{\prod _{k \in I}|C_k|}{|\ker (F_{I \setminus \{j\},j})|} \quad \text{ for } \text{ all } j \in I\text{. } \end{aligned}$$

We also have

$$\begin{aligned} \dim _{\mathbb {F}_{q^m}} \ker F_{I \setminus \{j\},j} = \sum _{k \in I\setminus \{ j\}} s_k - {\text{ rank }}(F_{I \setminus \{j\},j}). \end{aligned}$$

Therefore, taking the logarithm with base $q^m$ in Eq. (5) yields

6 $$\begin{aligned} {\text{ rank }}(F_{I,j}) \ge \alpha _1 + \ldots +\alpha _n -\sum _{k \in I\setminus \{j\}} s_k + {\text{ rank }}(F_{I \setminus \{j\},j}) \quad \text { for } \text { all } j \in I\text {, } \end{aligned}$$

which is the desired bound.

It remains to show that Claim A holds. We only prove it for $I=\{1,...,n\}$ and $j=1$, as the proof for all other cases is the same (but more cumbersome notation-wise). Fix an arbitrary $x \in C_1$ and view $F_{1,\{1,...,n\}}$ as an $\mathbb {F}_{q^m}$-linear map $\mathbb {F}_{q^m}^{s_1+ \cdots +s_n} \rightarrow \mathbb {F}_{q^m}^{t_1}$. Then $\text {Fan}_1(x,C)$ is the image of $C_2 \times \cdots \times C_n$ under the map

$$\begin{aligned} f: \mathbb {F}_{q^m}^{s_2 + \cdots +s_n} \rightarrow \mathbb {F}_{q^m}^{t_1}, \qquad (x_2,...,x_n) \mapsto (x,x_2,...,x_n) F_{\{1,...,n\},1}. \end{aligned}$$

We have $f= F_{11}x +g$ as functions, where

$$\begin{aligned} g: \mathbb {F}_{q^m}^{s_2 + \cdots +s_n} \rightarrow \mathbb {F}_{q^m}^{t_1}, \qquad (x_2,...,x_n) \mapsto (x_2,...,x_n) F_{\{2,...,n\},1}. \end{aligned}$$

Therefore, the images of *f* and *g* have the same cardinality, $|\text {Fan}_1(x,C)|$. Finally, by applying Lemma 22 to the $\mathbb {F}_{q^m}$-linear function *g*, we obtain

$$\begin{aligned} |\text {Fan}_1(x,C)| \cdot |\ker (g)| \ge |C_2 \times \cdots \times C_n|, \end{aligned}$$

which is the inequality in Claim A. $\square$

We illustrate Theorem 24 with two examples.

### Example 25

Let *q* be arbitrary and consider the *q*-LMUC $\mathcal {L}=(2,(2,2), (2,2),F)$, where

$$\begin{aligned} F = \begin{pmatrix} 1 &{} 0 &{} 0 &{} 0\\ 0 &{} 1 &{} 1 &{} 0\\ 0 &{} 1 &{} 1 &{} 0\\ 0 &{} 0 &{} 0 &{} 1 \end{pmatrix}. \end{aligned}$$

**Fig. 4:**
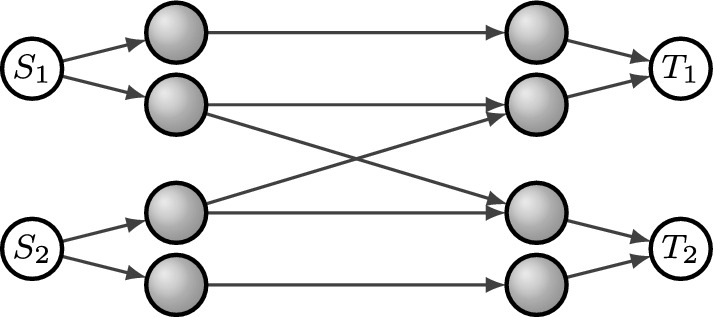
Network for Example 25

Note that $\mathcal {L}$ is induced, for example, by the network in Fig. 4.

By applying Theorem 24, we obtain that for all $m \ge 1$ and all $(\alpha _1,\alpha _2) \in \mathcal {R}_m(\mathcal {L})$ we have $\alpha _1 + \alpha _2 \le 3$, since

$$\begin{aligned} {\text {rank}}\begin{pmatrix} F_{11}\\ F_{21} \end{pmatrix} - {\text {rank}}F_{21} +s_2&= 2 - 1 + 2 = 3,\\ {\text {rank}}\begin{pmatrix} F_{12}\\ F_{22} \end{pmatrix} - {\text {rank}}F_{12} +s_1&= 2 - 1 + 2 = 3. \end{aligned}$$

For $m=1$, the 1-codes $C = \left( \mathbb {F}_q^2, \langle (0, 1)\rangle \right)$ and $C=\left( \langle (1,0)\rangle , \mathbb {F}_q^2\right)$ are both unambiguous, meaning that the rates (2, 1) and (1, 2) are achievable in one shot. This implies that the upper bound of Theorem 24 is tight in this case.

### Example 26

Let *q* be arbitrary and consider the *q*-LMUC $\mathcal {L}=(2,(1,2), (1,2),F)$, where

$$\begin{aligned} F = \begin{pmatrix} 1 &{} 1 &{} 1\\ 1 &{} 1 &{} 0\\ 1 &{} 0 &{} 1 \end{pmatrix}. \end{aligned}$$

**Fig. 5:**
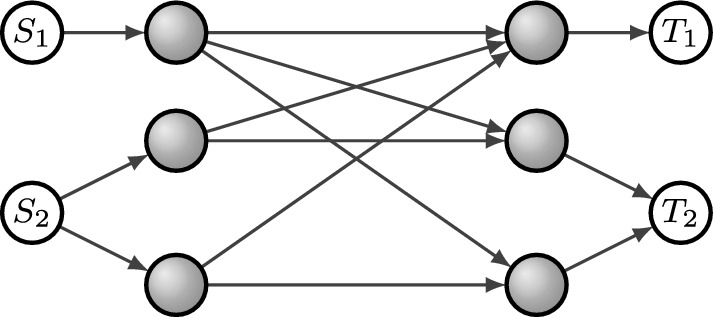
Network for Example 25

Note that $\mathcal {L}$ is induced, for example, by the network in Fig. 5.

By applying Theorem 24 we obtain that for all $m \ge 1$ and for all $(\alpha _1,\alpha _2) \in \mathcal {R}_m(\mathcal {L})$ we have $\alpha _1+\alpha _2\le 2$. Indeed,

$$\begin{aligned} {\text {rank}}\begin{pmatrix} F_{11}\\ F_{21} \end{pmatrix} - {\text {rank}}F_{21} +s_2&= 1 - 1 + 2 = 2, \\ {\text {rank}}\begin{pmatrix} F_{12}\\ F_{22} \end{pmatrix} - {\text {rank}}F_{12} +s_1&= 2 - 1 + 1 = 2. \end{aligned}$$

In the next section, we will show that whether or not this bound is sharp depends on the *characteristic* of the finite field $\mathbb {F}_q$ and not, for example, on *m*.

## The role of the field characteristic

The goal of this section is to illustrate the role that the field characteristic plays in the problem we are considering. Note that the problem we are studying in this section is an extension of the problem studied until now. When defining a *q*-LMUC, since *q* is a given parameter of the network, the characteristic of the field is, in general, fixed. The problem we consider in this section is based on the remark that any field contains the neutral elements for addition and multiplication and that in general those are denoted by 0 and 1. This implies that any matrix with entries only in $\{0,1\}$ can be the transfer matrix of a *q*-LMUC for any prime power *q*. It is natural to look into the achievable rates regions across different fields for these types of *q*-LMUCs.

It is well known that the size of the field plays an important role in achieving the network capacity for multicast networks when the network code design is part of the problem, see [2, 3]. We show that in sharp contrast with this scenario, the *characteristic* of the underlying field plays an important role in our model. Note that it is not the first time the characteristic of the field has played an important role in network coding. In [4], the authors show networks for which capacities are achievable in either even or odd characteristic fields. It is worth repeating that our problem differs from the one studied in [4] since the authors focus on constructing a network code, whereas in our case, the network code is frozen.

We show that given the *q*-LMUC from Example 26, the achievable rates region of the network over an odd characteristic field strictly contains the achievable rates region over an even characteristic field.

### Theorem 27

Let $\mathcal {L}=(2,(1,2),(1,2),F)$ be the *q*-LMUC from Example 26. Recall that

$$\begin{aligned} F= \begin{pmatrix} 1 &{} 1 &{} 1 \\ 1 &{} 1 &{} 0 \\ 1 &{} 0 &{} 1 \end{pmatrix}. \end{aligned}$$

If *q* is odd, then $(1,1) \in \mathcal {R}_1(\mathcal {L}) \subseteq \mathcal {R}(\mathcal {L})$. If *q* is even, then for any $m \ge 1$ and any $(\alpha _1,\alpha _2) \in \mathcal {R}_m(\mathcal {L})$ we have

7 $$\begin{aligned} 2\alpha _1+\alpha _2 \le 2. \end{aligned}$$

In particular, $$(1,1) \notin \mathcal {R}(\mathcal {L})$$.

### Remark 28

Note that this result not only proves that the achievability regions of the network when using even or odd characteristics are different, but it also shows that the achievability region when communicating using an even characteristic field is strictly contained in the one obtained using an odd characteristic field. More specifically, an interested reader will be able to see that, when using even characteristic fields, the rate (1, 1) not only is not achievable but is bounded away, meaning that it cannot be achieved even with infinitely many uses of the network.

### Proof

Observe that if *q* is odd, then the 1-code $C=\mathbb {F}_q\times \langle (1,-1)\rangle$ is unambiguous and therefore $(1,1) \in \mathcal {R}_1(\mathcal {L})$, as claimed.

To prove the second part of the theorem, denote by $f_{i,j}$ the multiplication by $F_{i,j}$ on the right, for $i,j\in \{1,2\}$. Let $(\alpha _1,\alpha _2) \in \mathcal {R}_m(\mathcal {L})$ and let $C=C_1\times C_2$ be an unambiguous *m*-code with $\log _{q^m}(C_1)=\alpha _1$ and $\log _{q^m}(C_2)=\alpha _2$. Recall that by Eq. (2), we have that $|\text {Fan}_1(x,C)|=|\text {IS}_1(C)|$ for all $x\in C_1$. Since *C* is unambiguous, we have $|C_1|\cdot |\text {IS}_1(C)|\le |\mathbb {F}_{q^m}|=q^m$, or equivalently

8 $$\begin{aligned} |\text {IS}_1(C)|\le \frac{q^{m}}{|C_1|}. \end{aligned}$$

Recall that, by definition, $\text {IS}_1(C)=f_{2,1}(C_2)$. Observe that for all $x\in \mathbb {F}_{q^m}$ we have $f_{2,1}^{-1}(x)=\{(y,x-y)\mid y\in \mathbb {F}_{q^m}\}$ and therefore it holds that

$$\begin{aligned} C_2\subseteq f_{2,1}^{-1}(f_{2,1}(C_2))=f_{2,1}^{-1}(\text {IS}_1(C))=\bigcup _{x\in \text {IS}_1(C)}f_{2,1}^{-1}(x)=\bigcup _{x\in \text {IS}_1(C)}\{(y,x-y)\mid y\in \mathbb {F}_{q^m}\}. \end{aligned}$$

In particular, all the elements of $C_2$ are of the form $(y,x-y)$ for some $x \in \text {IS}_1(C)$ and $y \in \mathbb {F}_{q^m}$. Now fix any $y\in \mathbb {F}_{q^m}$ and $x\in \text {IS}_1(C)$ with $(y,x-y)\in C_2$, and observe that

9 $$\begin{aligned} \text {Fan}_2((y,x-y),C)=f_{2,2}(y,x-y)+\text {IS}_2(C)=(y,x-y)+\langle (1,1)\rangle . \end{aligned}$$

Here is where the characteristic of the field starts to play a crucial role. If *q* is even, then Eq. (9) can be rewritten as

$$\begin{aligned} \text {Fan}_2((y,x+y),C)= (y,x+y)+\langle (1,1)\rangle =\{(z,x+z)\mid x\in \mathbb {F}_{q^m}\}=f_{2,1}^{-1}(x). \end{aligned}$$

It follows that

10 $$\begin{aligned} \bigcup _{(y,x+y)\in C_2}\text {Fan}_2((y,x+y),C)\subseteq \bigcup _{x\in \text {IS}_1(C)} f_{2,1}^{-1}(x)=f_{2,1}^{-1}(\text {IS}_1(C)). \end{aligned}$$

Again by Eq. (2) and that $f_{1,2}$ is injective, we have $|\text {Fan}_2((y,x+y),C)|=|\text {IS}_2(C)|=|C_1|$. Combining this fact with Eq. (10) and with the unambiguity of *C* we obtain

$$\begin{aligned} |C_2|\le \frac{|f_{2,1}^{-1}(\text {IS}_1(C))|}{|\text {Fan}_2((y,x+y),C)|}=\frac{|\text {IS}_1(C)|\cdot |\mathbb {F}_{q^m}|}{|C_1|}\le \left( \frac{q^m}{|C_1|}\right) ^2, \end{aligned}$$

where the last equality is a consequence of Eq. (8). Taking the logarithm with base $q^m$ of the inequality above, one gets $\alpha _2 \le 2(1-\alpha _1)$, as desired. $\square$

The following proposition shows that some of the rates satisfying Eq. (7) are achievable.

### Proposition 29

Let $\mathcal {L}=(2,(1,2),(1,2),\mathbb {F}_{2^m})$ be the $2^m$-LMUC from Example 26. Then for any $n\le m$ we have $\left( \frac{n}{m}, 2\left( 1-\frac{n}{m}\right) \right) \in \mathcal {R}(\mathcal {L})$.

### Proof

Let $\{x_1,\dots ,x_m\}$ be an ordered basis of $\mathbb {F}_{2^m}$ over $\mathbb {F}_2$. Define $C_1=\langle x_1,\dots ,x_n\rangle _{\mathbb {F}_2}$ and $C_2=\langle (x_i,0),(0,x_i)\mid i=n+1,\dots ,m\rangle _{\mathbb {F}_2}$. We can compute the interference sets $\text {IS}_1(C)=f_{2,1}(C_2)=\langle x_{n+1},\dots ,x_m\rangle _{\mathbb {F}_2}$ and $\text {IS}_2(C)=f_{1,2}(C_1)=\langle (x_1,x_1),\dots ,(x_n,x_n)\rangle _{\mathbb {F}_2}$. Thus $C_1\times C_2$ is unambiguous because $C_1\cap \text {IS}_1(C)=C_2\cap \text {IS}_2(C)=\{0\}$. $\square$

## Conclusions

We considered the multiple unicast problem over a coded network where the network code is linear over a finite field and fixed. We introduced a framework to define and investigate the achievable rate region in this context. We established an outer bound on the achievable rate region and provided examples where the outer bound is sharp. Finally, we illustrated the role played by the field characteristic in this problem, which is different than what is generally observed in the context of linear network coding.

## Data Availability

Data sharing not applicable to this article as no datasets were generated or analysed during the current study.
